# Interdomain Interactions Modulate Refolding Kinetics and Aggregation in a Monoclonal Antibody

**DOI:** 10.1021/jasms.5c00166

**Published:** 2025-08-26

**Authors:** Philipp Trolese, Andrea Pierangelini, Benedetta Fongaro, Patrizia Polverino de Laureto

**Affiliations:** † Department of Pharmaceutical and Pharmacological Sciences, University of Padova, Padova 35131, Italy; ‡ Department of Neuroscience, Biomedicine and Movement Sciences, University of Verona, Verona 37134, Italy

**Keywords:** monoclonal antibodies, hydrogen−deuterium exchange mass spectrometry, aggregation, unfolding, refolding

## Abstract

Understanding the structural determinants of antibody stability and aggregation is essential for therapeutic development. In this study, we investigated the unfolding and refolding behavior of bevacizumab under denaturing conditions using dynamic light scattering (DLS), circular dichroism (CD), and hydrogen–deuterium exchange mass spectrometry (HDX-MS). Unfolding was induced by incubating the antibody in 4 M guanidine hydrochloride (Gnd-HCl), followed by refolding through dilution with 1 M Gnd-HCl. Each domain exhibited distinct unfolding kinetics: the C_H_2 and V_H_ domains unfolded rapidly, while the C_H_3 domain retained its structure until 45 min, consistent with its known thermodynamic stability. Aggregation, detected by DLS, was prevalent only after 120 min and overnight unfolding, coinciding with C_H_3 destabilization. Notably, aggregation-prone regions were identified in both the Fc and Fab portions of the antibody. Specifically, interactions at the C_H_2–C_H_3 and C_H_3–C_H_3 interfaces appear disrupted upon C_H_3 unfolding, leading to misfolded and aggregation-prone states in both domains. In parallel, the V_H_ CDR H1 region exhibited aberrant protection after refolding, suggesting its involvement in aggregation. These findings highlight the cooperative nature of C_H_2–C_H_3 refolding and underscore the critical role of the C_H_3 stability in preventing aggregation. The involvement of both constant and variable domains emphasizes the complex, interdependent nature of monoclonal antibody aggregation. This work provides mechanistic insights into domain-specific contributions to folding and aggregation, offering guidance for the design of more stable therapeutic antibodies.

## Graphical abstract

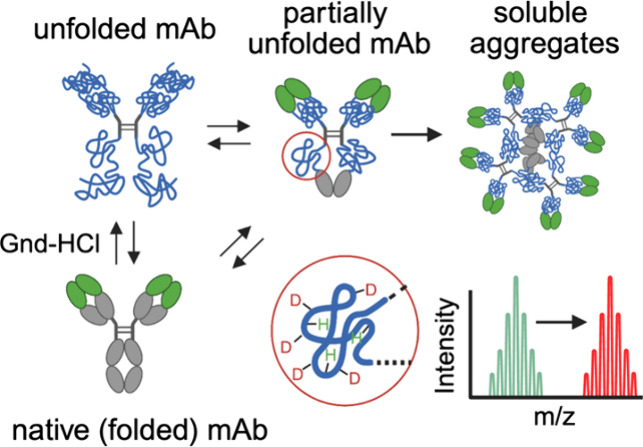

## Introduction

Monoclonal antibodies (mAbs) and their fragments represent the largest and most rapidly expanding class of biopharmaceuticals.^1^ A major challenge during their development, manufacturing, and storage is protein aggregation, which could compromise both the efficacy and safety of the final product.^2^ Carpenter et al. raised the concern about overlooking subvisible particles,^3^ and as such, regulatory authorities require formulations with minimal aggregate levels throughout shelf life^4^ because even small amounts of aggregates may trigger immune responses or reduce therapeutic potency.^5,6^ Understanding the mechanisms underlying antibody aggregation is thus critical to developing effective mitigation strategies for mAbs degradation.

Evidence suggests that protein aggregation often proceeds through partially unfolded intermediates, rather than from native or fully denatured states.^7,8^ Multidomain proteins, such as mAbs, may also aggregate via the unfolding of one or more domains.^9,10^ In this study, we use bevacizumab, a recombinant humanized IgG1 monoclonal antibody, as a model to explore the domain-level contributions to aggregation. Bevacizumab is an anti-VEGF mAb used in oncology and off-label in ophthalmology,^11^ with a molecular weight of ∼149 kDa and an isoelectric point of 8.3,^12^ exhibiting high thermodynamic stability at pH 6.^13,14^ It has a Y-shaped structure with two identical light chains (V_L_-C_L_) and two identical heavy chains (V_H_-C_H_1-C_H_2-C_H_3), stabilized by 16 disulfide bridges.^15^ The stem of the Y makes the Fc region composed of a C_H_2-C_H_3 dimer. A glycan at position Asn303 fills the space between the C_H_2 domains, whereas extensive protein–protein interactions are present between the opposing C_H_3 domains. The arms of the Y shape composed by a dimer between the light chains and V_H_-C_H_1 make up the antigen binding fragment (Fab) and provide two binding sites for VEGF.^16^

Recently, it has been shown that exposure to low pH, high ionic strength, and freeze and thaw cycles can stimulate aggregation of mAbs by favoring the formation of partially unfolded protein molecules.^17–20^ For instance, low pH is used for protein A chromatography and viral inactivation. It was found that exposure of a mAb to pH 2 led to the formation of scarcely soluble aggregates and fragmentation when taken back to neutral conditions.^21^ Both processes were even exacerbated by exposure to light. Studies investigating antibody aggregation under pharmaceutically relevant stress conditions have also sought to identify specific protein domains that unfold and drive the aggregation process. In fact, Kim et al. demonstrated that Fab unfolding under low pH initiated the aggregation of IgG1 molecules,^22^ while Majumdar et al. found that aggregation induced by high salt concentrations was mediated by instability of the C_H_2 domain.^23^ Similarly, Carpenter et al. showed that exposure to low concentrations of guanidine hydrochloride (Gnd-HCl) led to selective unfolding of the C_H_2 domain, resulting in aggregation over time.^24^ Studies examining isolated constant domains further revealed that each domain exhibits distinct conformational and colloidal stabilities, suggesting that these intrinsic differences contribute to the propensity of an antibody to aggregate.^25^ However, a detailed understanding of domain-specific contributions to aggregation in therapeutic mAbs remains incomplete. Moreover, antibody aggregation is greatly heterogeneous and can vary substantially. Mechanisms of unfolding and aggregation are strongly influenced by the specific destabilizer. Of note, the aggregation propensity of a protein is determined not only by environmental factors but also by its inherent structural and physicochemical properties.^26^ Furthermore, aggregate morphology varies greatly, encompassing covalent and noncovalent, reversible and irreversible, as well as soluble and insoluble forms, with sizes ranging from 50 to 3000 nm, according to Vázquez-Rey.^2^

To examine bevacizumab aggregation caused by partially unfolded states, we induced protein denaturation using guanidine hydrochloride (Gnd-HCl), followed by refolding through dilution. The Gnd-HCl-induced process correlates especially with a destabilizing effect on protein electrostatic interactions.^27^ We hypothesized that partially unfolded states transiently populated during refolding may drive aggregation. To test this, we used a combination of biophysical techniques, including hydrogen–deuterium exchange mass spectrometry (HDX-MS), dynamic light scattering (DLS), and circular dichroism (CD) spectroscopy. HDX-MS provides a fast, accurate, and detailed method to assess protein structure and dynamics. As HDX-MS can measure backbone flexibility at a resolution of 5–20 residues,^28^ numerous regulatory bodies have previously approved it as a structural technique for the validation of protein biopharmaceuticals, including medications based on antibodies, and it is widely acknowledged as an essential tool.^29^ It is especially crucial to take flexibility into consideration for understanding the physicochemical stability of a protein, including its aggregation propensity.^30^ For instance, HDX-MS was already used to investigate effects of salts on the conformational stability and aggregation of antibodies.^23^

In this work, we aim to systematically characterize domain-specific unfolding events driving aggregation in bevacizumab using a combination of HDX-MS and complementary biophysical methods. By identifying instabilities in specific domains, our findings could help in the design and formulation of more stable therapeutic antibodies. Alterations in deuterium uptake profiles may direct targeted engineering efforts toward structurally vulnerable regions and aid the development of tailored formulation strategies aimed at mitigating aggregation driven by partial unfolding.

## Experimental Section

### Materials

Bevacizumab (Oyavas) was provided by a hospital pharmacy as fresh daily residues after patient treatments at a concentration of 25 mg/mL. Tris­(2-carb­oxy­ethyl)­phos­phine (TCEP), guanidine hydrochloride (Gnd-HCl), and other reagents were provided by Merck (Darmstadt, Germany).

### Sample Preparation

For the unfolding experiments, the mAb was taken from the vial and diluted in 7 M guanidine hydrochloride (Gnd-HCl) in 20 mM sodium phosphate at pH 7.4 to achieve a final concentration of 16 μM in 4 M Gnd-HCl. The concentration of 4 M Gnd-HCl was chosen based on its ability to fully denature mAbs^31^ with respect to lower concentrations that seem to denature only the least stable domains.^24^ The sample was incubated for 2, 10, 45, and 120 min and overnight (ON) before analyses. To investigate refolding from partially unfolded states, the mAb was first diluted in 7 M Gnd-HCl to a final concentration of 64 μM in 4 M Gnd-HCl and incubated for various time points. It was then diluted with 20 mM sodium phosphate buffer (pH 7.4) to reduce the Gnd-HCl concentration to 1 M, initiating the refolding process. The refolding incubation times matched the unfolding times: i.e., the mAb was unfolded for 2 min in 4 M Gnd-HCl and then allowed to refold for 2 min in 1 M Gnd-HCl, and so on for the remaining time points. For refolding from the fully denatured state, the mAb was incubated overnight in 4 M Gnd-HCl at a concentration of 64 μM. Following this, it was diluted with 20 mM sodium phosphate buffer (pH 7.4) to a final Gnd-HCl concentration of 1 M. The sample then underwent a second incubation for 2, 10, 45, and 120 min and overnight, respectively, before being immediately analyzed.

### Dynamic Light Scattering (DLS)

DLS analysis was conducted with a Zetasizer Ultra instrument (ZSU5700, Malvern instruments, Worcestershire, UK). Scattering data were analyzed with the ZS Xplorer software and expressed by intensity. The appropriate attenuator position was automatically determined by the Zetasizer instrument during the measurement sequence. The mean count rate was between 250 and 350 kcps in all measurements. UV-transparent disposable cuvettes with a 0.45 cm path length (Sarstedt, Nümbrecht, Germany) were employed. The analysis was conducted in triplicate. In all measurements, the concentration of bevacizumab was kept at 1 mg/mL. DLS data are reported as a number distribution, and the values are corrected for the viscosity of 1 and 4 M Gnd-HCl listed in Kawahara et al.^32^ The percentage of high molecular weight (HMW) species is calculated by adding the percentages of all species with a diameter bigger than that of the monomer.

### Spectroscopic Measurements

Protein concentrations were determined by absorption measurements at 280 nm using a double-beam Lambda-20 spectrophotometer (PerkinElmer Life Sciences). The molar absorptivity at 280 nm for bevacizumab was 1.66 mL mg^–1^ cm^–1^, as evaluated from its amino acid composition by the method of Gill and von Hippel.^33^ The secondary and tertiary structure content of the proteins was assessed by far- and near-UV circular dichroism (CD), respectively. The measurements were performed on a J-800 Series spectropolarimeter (JASCO, Tokyo, Japan) in a 0.1 mm cylindrical quartz cuvette for the far-UV CD measurements and in a 5 mm rectangular quartz cuvette for the near-UV CD measurements. In both cases, the concentration was 0.5 mg/mL. Data were recorded in the wavelength range of 250–195 nm and 330–250 for far- and near-UV CD, respectively, collecting data with high-tension voltage < 600 V and avoiding noisy signals. All samples were measured at the same settings averaged in three and the buffer data subtracted. The mean residue ellipticity [θ] (deg cm^2^ dmol^–1^) was calculated from the formula [θ] = (θ_obs_/10)­(MRW/*lc*), where θ_obs_ is the observed ellipticity in degrees; MRW is the mean residue molecular weight of the protein; *l* is the optical path length in cm; and *c* is the protein concentration in g/mL. The spectra were recorded in 20 mM sodium phosphate buffer pH 7.4 in the presence of a specific concentration of Gnd-HCl.

### Hydrogen–Deuterium Exchange Mass Spectrometry (HDX-MS) Measurements

HDX-MS measurements were performed using a Xevo G2S Q-TOF (Waters) mass spectrometer equipped with a standard electrospray ionization source, an Acquity M-class UPLC (Waters), and an Automation 2.0 sample workstation (Waters). The final concentration of mAb before the addition of D_2_O was 16 μM. For the H/D exchange reaction, an aliquot (2.5 μL) of each sample was diluted 10-fold in deuterated buffer (20 mM sodium phosphate, pD 7.0, in 99.9 % D_2_O) and was then allowed to exchange for 10 s at room temperature. H/D exchange was quenched at 0 °C by a 2-fold dilution in quenching buffer (7 M Gnd-HCl, 0.5 M TCEP, 0.8 % formic acid pH 2.15) for 1 min on ice to aid the protein denaturation. Successively, the samples were diluted 2-fold with 0.8 % formic acid to a final Gnd-HCl concentration below 2 M, and 100 μL of the solution was analyzed (approximately 50 pmol of mAb). The effluent was analyzed by a Xevo G2S Q-TOF mass spectrometer (*m*/*z* 50–2000), and each peptic fragment was identified by the MS^E^ mode. The deuterium uptake values were expressed as the percentage of differential deuterium uptake and calculated as follows: % Δ*D* = (*U*_t_ – *U*_0_)/(*U*_max_ – *U*_0_), where *U*_t_ is the uptake of the fragments at each time point, and *U*_0_ and *U*_max_ are the uptakes of the nonincubated fragments and the maximally deuterated samples (maxD), respectively. These values refer to the difference in uptake between the samples treated with Gnd-HCl and the folded (nonincubated) samples, as clarified in each condition. The maximally deuterated samples used for back-exchange corrections were prepared according to Peterle et al.^34^ Fragments generated from on-line pepsin digestion were identified using the Protein Lynx Global Server 3.0 and then analyzed with DynamX 3.0 software (Waters). For the analysis, only fragments matching the following criteria were considered: (i) a 5% retention time window in the chromatographic separation; (ii) a maximum MH^+^ error of 6 ppm; (iii) at least 2 ion products identified for each peptic fragment; (iv) a minimum of 0.3 ion products generated per amino acid in the fragment; (v) fragments containing 33 amino acids were excluded due to identification ambiguity and poor sequence localization. Data are presented as the mean of biological triplicates for most measurements with some points based on duplicates. Welch’s *t* test was performed to determine the statistical significance, using *p* = 0.05 as a threshold.

## Results and Discussion

### Unfolding of Bevacizumab by Guanidine Hydrochloride

To identify aggregation-prone regions within the monoclonal antibody bevacizumab, we performed HDX-MS measurements alongside circular dichroism (CD) and dynamic light scattering (DLS) to monitor structural changes during chemical unfolding and refolding. Bevacizumab domains were subdivided by visual inspection using two PDB structures: the crystal structure of its Fab fragment (PDB ID: 7V5N) and the crystal structure of human IgG1-Fc that shows ∼99% sequence identity with bevacizumab (PDB ID: 5JII). As such, the numbering of residues is based specifically on the sequence of bevacizumab and may not correspond fully with the numbering of the deposited structure for the Fc. In Figure 1A–C, the characterization of the mAb by DLS and CD was reported. DLS showed an increase of the diameter of the mAb from ∼10 nm (native condition) to ∼15 nm upon ON incubation in 4 M Gnd-HCl. This increase may have resulted mainly from unfolding and/or the formation of a small number of aggregates. As a reference, Mehta et al.^24^ reported the solubilization of formed mAb aggregates already at 2 M Gnd-HCl. In parallel to the increase in size, Figure 1B,C reveals a time-dependent denaturation of the secondary and tertiary structures of the mAb incubated in the presence of this amount of denaturant. By HDX-MS analysis, it was evidenced that each domain of bevacizumab exhibits distinct unfolding behaviors in 4 M Gnd-HCl (Figure 1D). The percentage of differential deuterium uptake was evaluated from the difference between untreated (no Gnd-HCl) and fully unfolded samples (4 M Gnd-HCl). The V_L_ domain unfolds gradually over time, while the V_H_ and C_H_2 domains reach maximal unfolding almost immediately. In contrast, the C_H_3 domain maintains its native structure the longest and undergoes a delayed, burst-like unfolding after approximately 45 min. This observation is consistent with the literature describing C_H_2 as the least stable and C_H_3 as the most stable domain, due to extensive interdomain interactions.^31,35^ Figure 1E shows the differential deuterium uptake for C_H_3 with greater resolution. The whole sequence is shown in Figure S1. White gaps indicate regions with no coverage in the HDX-MS experiments. Interestingly, two specific areas seem to be the most resistant to unfolding: ∼360–380 and ∼405–430. By analyzing the crystal structure of the Fc domain, these two stretches of amino acids appear to be located at the interface between the two C_H_3 domains and hence host extensive protein–protein interactions. Thus, the initial resistance of the C_H_3 domain to unfolding may be due to these interactions. As a remark, even after overnight incubation of the mAb in 4 M Gnd-HCl, a little residual structure possibly due to disulfide bonds persists in comparison to the maximally deuterated protein, which is obtained under reducing conditions. Notably, disulfide bonds have been shown to affect the stability and unfolding reversibility of mAbs.^36^

**Figure 1:**
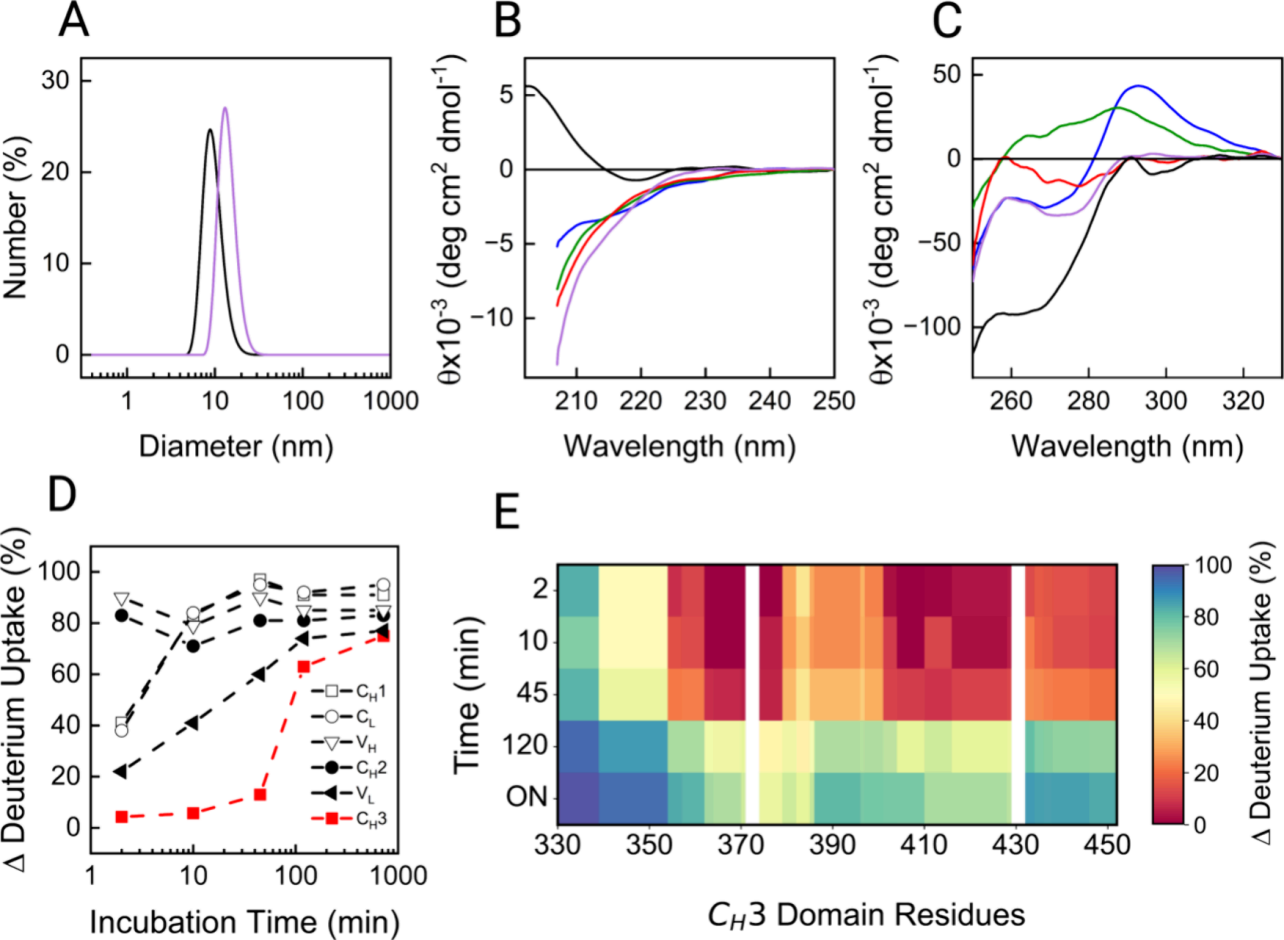
Unfolding of bevacizumab in 4 M Gnd-HCl probed by DLS (A) and far-UV (B) and near-UV (C) CD experiments. Each trace represents a specific incubation time point: T0 (black), 2 min (blue), 45 min (green), 120 min (red), and overnight (ON; purple). (D) The mean percentage of differential deuterium uptake of each domain is plotted against the incubation time. (E) The percentage of differential deuterium uptake between the unfolded and native states for all residues in the C_H_3 domain is shown as a heatmap for all time points. Adjacent to this is the color code for the heatmap, with red being 0 % uptake and blue being 100 % uptake.

### Refolding of Bevacizumab from Its Partially Unfolded State

Refolding from partially unfolded states was achieved by incubating the mAb in 4 M Gnd-HCl for increasing time, followed by a matching-duration refolding step in 1 M Gnd-HCl, a condition that does not alter the bevacizumab structure and behavior (Supporting Information, Figure S2). Structural recovery was monitored using DLS, CD, and HDX-MS (Figure 2). DLS analysis revealed that no sample was able to recover the hydrodynamic diameter of the native mAb. Samples incubated for 2, 10, and 45 min exhibited a diameter of ∼16, ∼14, and ∼17 nm, respectively, after refolding (Figure 2A). These diameters mostly fit with the diameter of the unfolded mAb after ON incubation in 4 M Gnd-HCl. Deviations from this value may be due to the presence of a small number of aggregates that skews the diameter toward larger values. Hence, the main species found in solution should be the misfolded monomer. Samples incubated for 120 min display a diameter of ∼22 nm, indicative of a significant presence of small oligomers. Samples subjected to overnight unfolding followed by overnight refolding (purple lines) exhibited the largest increase in hydrodynamic diameter (from ∼10 to ∼45 nm), reflecting the presence of large oligomers. Interestingly, when the sample unfolded for 120 min is subjected to ON refolding, it reaches a size of ∼32 nm (Supporting Information, Figure S3), suggesting that the formation of large oligomers is accelerated by ON unfolding. Of note, samples with a prevalence of oligomers were observed only when the C_H_3 domain has been unfolded (120 min and ON). CD analysis of the refolded samples (Figure 2B,C) indicated that under all conditions, the protein was able to recover its secondary and tertiary structures to some extent with slight differences. In some of these samples, aggregates were present, especially after 120 min and ON. It is possible to infer that these aggregates exhibit native-like secondary and tertiary structures to some extent, which may have formed after partial refolding of the protein.

**Figure 2:**
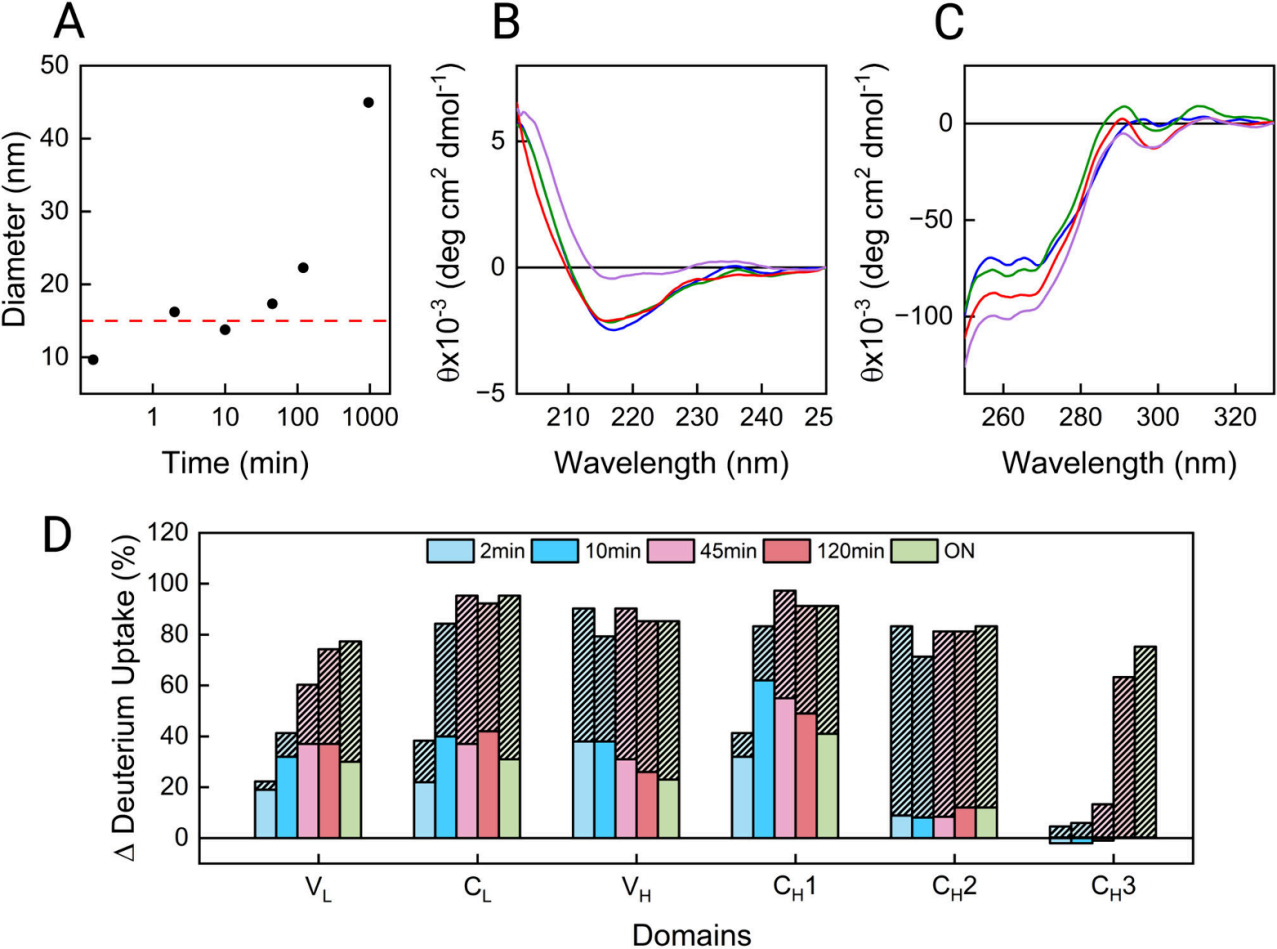
Refolding of bevacizumab from partially folded states studied by DLS (A) and far-UV (B) and near-UV (C) CD spectroscopy. Samples were incubated in 4 M Gnd-HCl for unfolding, followed by dilution to 1 M Gnd-HCl for refolding; the incubation times in both steps were matched. In (A), the mean diameter obtained from the number distribution by DLS is plotted against incubation time points. The horizontal red dashed reference line at about 15 nm represents the unfolded mAb after ON incubation in 4 M Gnd-HCl. In (B) and (C), each trace represents a specific incubation time point: 2 min (blue), 10 min (orange), 45 min (green), 120 min (red), and overnight (ON; purple). (D) Bar plot reporting the mean differential deuterium uptake for each domain compared with the native state. The shaded columns represent the differential deuterium uptake after unfolding in 4 M Gnd-HCl for a specific time. The nonshaded colored columns represent the differential deuterium uptake after refolding in 1 M Gnd-HCl for the same amount of time.

Figure 2D shows the mean differential deuterium uptake after refolding for each domain with respect to the uptake after unfolding (shaded bars), both compared to the native state. The C_H_1 domain showed the least refolding efficiency, while C_L_, V_L_, and V_H_ exhibited a partial recovery. C_H_2 and C_H_3 are the domains with the largest difference in deuterium uptake between the matching unfolding and refolding steps. Of note, the C_H_2 domain unfolded rapidly, even after 2 min in 4 M Gnd-HCl (Figure 1D), and seemed to refold with similar speed upon dilution, indicating a fast rate of unfolding and refolding. The C_H_3 domain only exhibited structural loss after ≥45 min in denaturant but seemed to efficiently recover its structure after refolding even in those cases. On the other hand, as we know that to some extent aggregates are present in all samples (but prevalent only after 120 min and ON), it is difficult to differentiate if the decrease in differential deuterium uptake is due to refolding or aggregation by only looking at the mean deuterium uptake for each domain. Figure 3 shows the differential deuterium uptake compared with the native state after refolding for the whole protein sequence. Gaps indicate sequences with no coverage in HDX-MS experiments (Supporting Information, Scheme S1).

**Figure 3:**
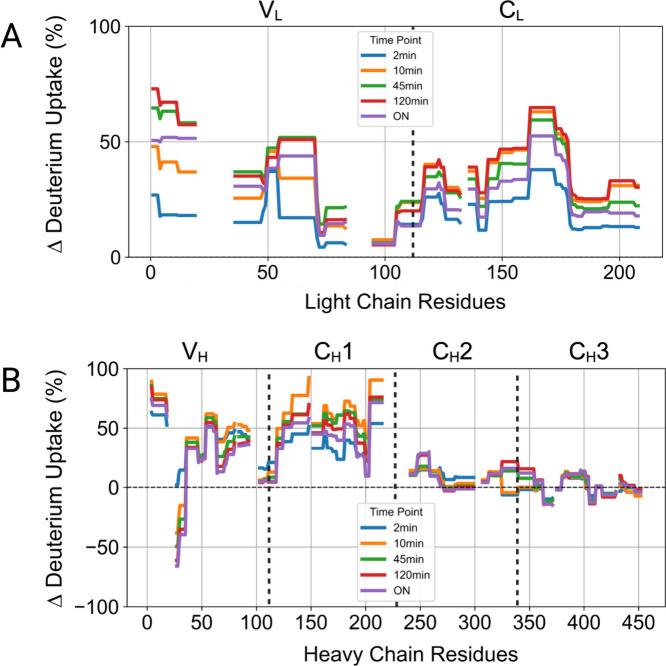
Differential deuterium uptake following refolding from partially unfolded states compared to the native state for the full mAb sequence at each time subdivided into light (A) and heavy (B) chains. Each trace represents one incubation time point: 2 min (blue), 10 min (orange), 45 min (green), 120 min (red), and ON (purple). Samples were first incubated in 4 M Gnd-HCl for unfolding and then diluted to 1 M Gnd-HCl for refolding; incubation times in both steps were matched. Black vertical dashed lines subdivide each chain into its domains.

Several regions displayed incomplete refolding even after a short unfolding time (e.g., 2–45 min), explaining why in DLS experiments the native diameter was never recovered. In the light chain, no region exhibits a negative deuterium uptake, which would indicate aberrant refolding or sequestration of that region in the aggregates. Some regions appear to refold well (e.g., ∼70–100), whereas others persist in a misfolded conformation. Notably, the C_H_2 and C_H_3 domains appeared to refold well in average, though with internal variability among peptides.

Residues 247–258 in the C_H_2 domain failed to refold properly only after 120 min and overnight unfolding. As commented above, these conditions caused significant prevalence of oligomers in the samples. When analyzing the crystal structure of the Fc, these regions were found to be located at the C_H_2–C_H_3 interface. On the other hand, some regions were identified to exhibit a lower deuterium uptake (363–370 and 405–410; 417–428 is not significant) than the native protein reaching the most negative value at high incubation times, linking them to aggregation. Of note, these regions are found at the C_H_3–C_H_3 interface. Residues 27–35 in the V_H_ domain, corresponding to the CDR H1 region, exhibit the biggest degree of protection with a differential deuterium uptake ranging from about −30 % after 10 min to about −70 % after ON incubation.

### Refolding of Bevacizumab from Its Fully Unfolded State

Focusing on the refolding pathway from the fully denatured state (Figure 4), we observed a gradual increase in hydrodynamic diameter (Figure 4A) with a significant prevalence of oligomers from 45 min onward, as the percentage of high molecular weight (HMW) species present in solution shows, calculated from the DLS number distribution (Figure 4B). This increase in size is accompanied by an apparent recovery of the secondary and tertiary structures over time (Figure 4C,D) as before, suggesting oligomers may contain monomers with native-like secondary and tertiary structure elements. At both 120 min and following overnight refolding, HMW species dominate the number-weighted distribution (∼100%). Notably, the increase in hydrodynamic diameter between these two time points suggests ongoing oligomer growth and/or reorganization.

**Figure 4:**
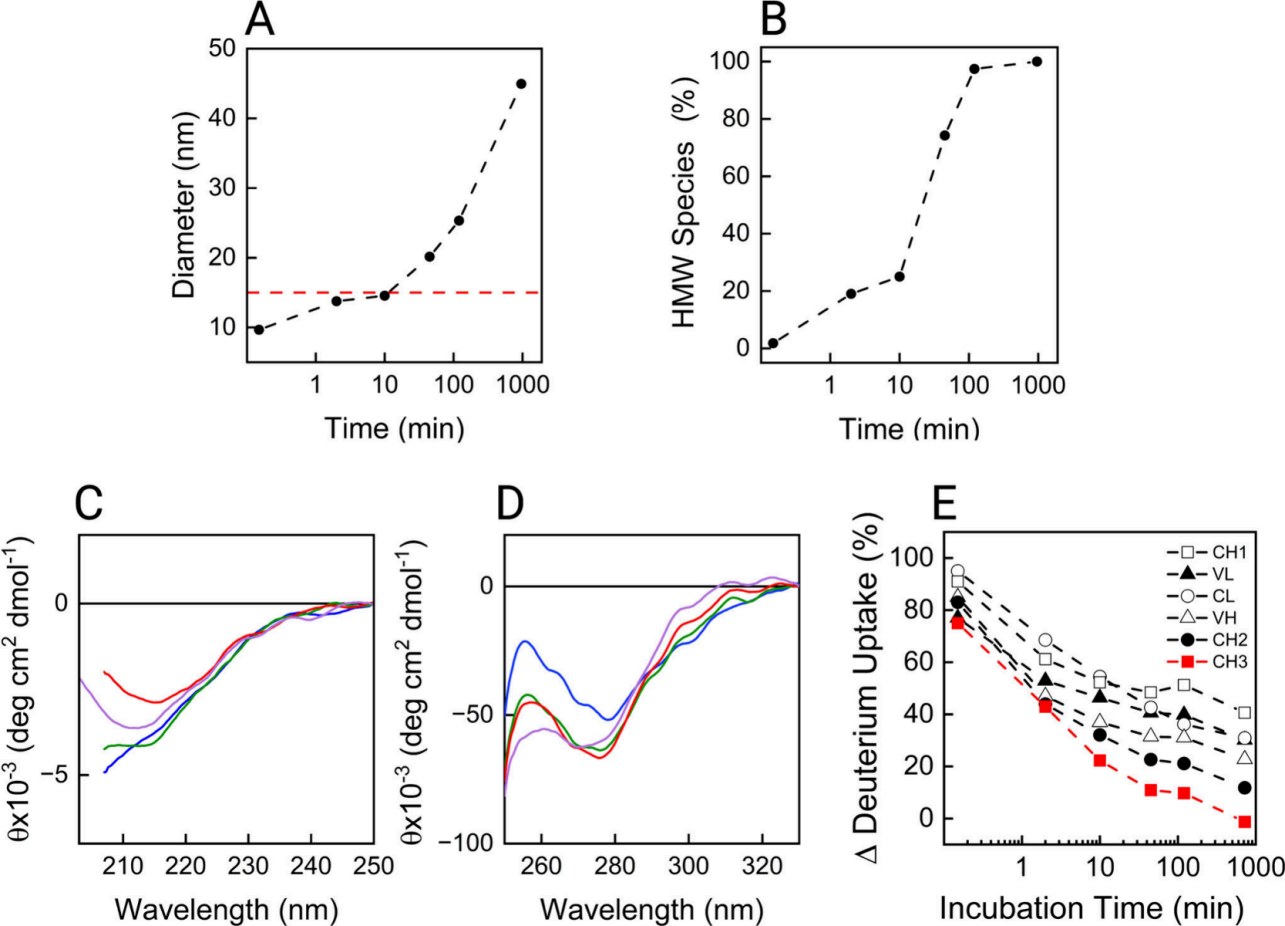
Refolding of the mAb from a fully denatured state studied by DLS (A, B), far-UV (C) and near-UV CD spectroscopy, and HDX-MS (E). Samples were first incubated ON in 4 M Gnd-HCl and then diluted to 1 M Gnd-HCl for refolding. The measurements were performed after each time point. Panel (A) shows the mean hydrodynamic diameter obtained from the number distribution plotted against refolding time. The horizontal red dashed line at ∼15 nm represents the diameter of the unfolded mAb incubated in 4 M Gnd-HCl ON. Panel (B) shows the percentage of high molecular weight (HMW) species (or oligomers) obtained from the number distribution plotted against refolding time. In (C) and (D), each trace represents a specific time point: 2 min (blue), 10 min (orange), 45 min (green), 120 min (red), and overnight (ON; purple). (E) The mean differential deuterium uptake of each domain is plotted against the incubation time.

HDX-MS analysis (Figure 4E and Figure S4) confirmed that the C_H_2 and C_H_3 domains show the smallest difference in deuterium uptake with the native state after ON refolding, indicating their involvement in misfolding and aggregation. The C_H_1 domain showed the least refolding across all conditions, and other domains failed in fully regaining native structure too. Interestingly, the C_H_3 domain recovers a great amount of structure already after 10 min of refolding (little aggregation), while the C_H_2 domain exhibits a delayed refolding. This is particularly notable, as C_H_2 previously showed rapid refolding kinetics (Figure 2D and Figure 3B). This delay suggests that unfolding and refolding of C_H_3 may slow C_H_2 refolding. In turn, C_H_2 might persist longer in a misfolded state, ultimately leading to significant aggregation in the samples after 10 min.

In Figure 5A, refolding from the fully unfolded state of some regions in the C_H_2 (left) and C_H_3 (right) domains is shown. The region 247–258 stalled at about 40 % differential deuterium uptake after 45 and 120 min refolding, apparently reaching a plateau. After ON refolding, the differential uptake jumped to 25 %. As commented before, in all samples after 10 min, aggregated species are prevalent, and from 120 min to ON, a big increase in diameter was observed. Thus, this region might be involved in the formation of larger oligomers after an initial misfolding. When examining the crystal structure of the Fc of an IgG antibody that has about 99 % sequence identity with that of bevacizumab, the region 247–258 appears in spatial proximity to the C_H_3 domain (Figure 5B). The insertion in Figure 5B specifically shows that residue L257 in the C_H_2 domain forms a hydrogen bond with residue H431 in the C_H_3 domain, revealed by the Residue Interaction Network Generator (RING).^37^ Additionally, the interface is stabilized by extensive van der Waals interactions involving L257, H431, and E426. This suggests that C_H_3 unfolding may impair C_H_2 refolding, either directly or through the disruption of stabilizing interdomain interactions. When the C_H_3 domain becomes unfolded, these are likely disrupted and/or may not re-establish correctly upon refolding, causing the formation of partially folded species that tend to aggregate. Negative differential deuterium uptake compared to the native state is observed for residues 363–370 and 405–410 in the C_H_3 domain. They are located at the C_H_3–C_H_3 interface and are highlighted in red in the structure in Figure 5A and B. Analysis with RING reveals extensive protein–protein interactions in the native state in these regions. These interactions are responsible for the high stability and late unfolding of the C_H_3 domain. On the other hand, failure to reconstitute these stabilizing interfaces could lead to misfolded regions in both the C_H_2 and C_H_3 domains that predispose the antibody to aggregation. Beyond the C_H_2–C_H_3 and C_H_3–C_H_3 interfaces, our data revealed another region potentially involved in aggregation: residues 25–35 in the V_H_ domain, corresponding to the CDR H1 loop. Figure 5C shows the decrease in differential deuterium uptake during refolding from the fully denatured state and the location of the CDR H1 loop in the crystal structure of bevacizumab Fab (PDB ID: 7V5N). Compared to the native state, this area exhibited a differential deuterium uptake of about −70 % after ON refolding, indicating strong protection. Of note, this region displays a differential deuterium uptake of −25 % even after 10 min refolding. DLS measurements estimated that at this time point, oligomers are not prevalent, suggesting that the observed protection at 10 min is unlikely to result from oligomerization. Instead, this may reflect local misfolding or compaction within the CDR H1 loop. Given that this region is highly solvent exposed in the native structure, even subtle misfolding could reduce the level of deuterium exchange relative to the native state. Nevertheless, after 10 min, where aggregates are significantly present, the CDR H1 region continues to lower its differential deuterium uptake, supporting a hypothesis that it initially misfolds and rigidifies. Subsequently, it gets involved in aggregation.

**Figure 5:**
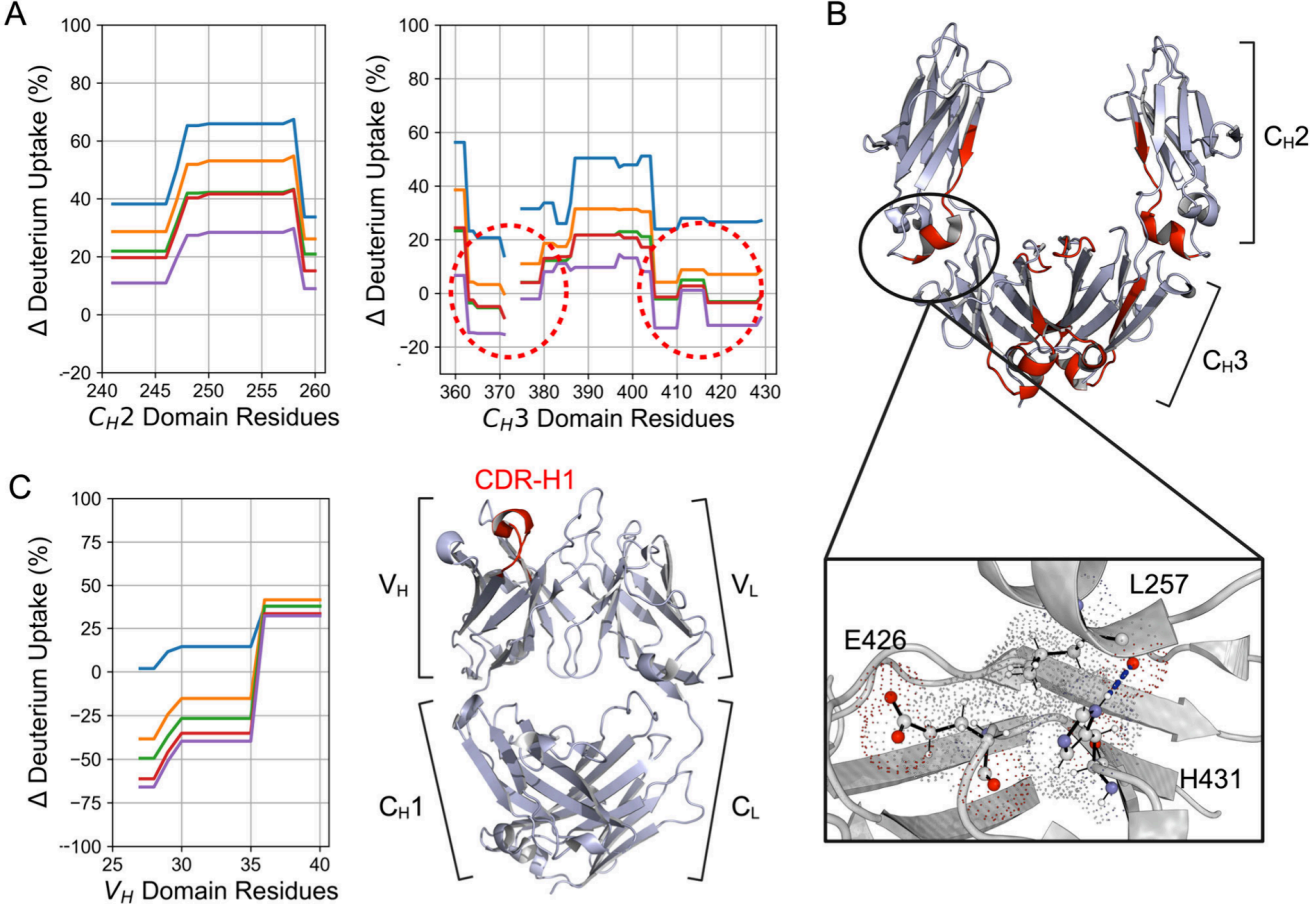
(A) Percentage of differential deuterium uptake compared to the native state of regions in the C_H_2 (left) and C_H_3 (right) domains during refolding from the fully denatured state. Each trace corresponds to a specific time point: 2 min (blue), 10 min (orange), 45 min (green), 120 min (red), and overnight (ON; purple). (B) 3D structure of the Fc region of an IgG (PDB ID: 5JII); the regions highlighted in red are believed to play a role in aggregation: residues 247–258 in the C_H_2 domain and 363–370 and 405–410 in the C_H_3 domain circled in panel (A). Below is a close-up view of the highlighted region, showing interactions between the C_H_2 and C_H_3 domains. These include van der Waals interactions (depicted as dotted surfaces) and a hydrogen bond between L257 in the C_H_2 domain and H431 in the C_H_3 domain. (C) Percentage of differential deuterium uptake of the portion in the V_H_ domain corresponding to the CDR H1 region, highlighted in red in the adjacent Fab structure of bevacizumab (PDB ID: 7V5N).

## Conclusions

The majority of proteins contain multiple domains. Their ability to fold or unfold depends on several factors, as determined by the environment and their intrinsic physicochemical properties. Sometimes the folding of a domain affects the stability and the folding of the close vicinal domain due to substantial native interactions between them. The interruption of these interactions can have a deep impact on local conformation, leading to exposure of regions of the protein normally hidden and, therefore, to aggregation.

Here, we report a model in which aggregation-prone regions span both the Fc and Fab portions of bevacizumab. Specifically, C_H_3–C_H_3 and C_H_3–C_H_2 interfaces, which contain extensive protein–protein interactions, appear to modulate the refolding kinetics of mAb. Notably, C_H_2 alone refolds rapidly; however, in the presence of an unfolded C_H_3 domain, C_H_2 refolding is delayed, potentially increasing the window of exposure for partially folded intermediates prone to aggregation. Interactions between C_H_2 and C_H_3 domains may play a stabilizing role, potentially mitigating aggregation by facilitating or accelerating the overall refolding process. Concurrently, aberrant protection in the V_H_ CDR H1 region implicates the variable domain in aggregation processes and oligomer growth. This could have therapeutic relevance because of the central role of CDRs in the binding of antigens.

Given the importance of domain interfaces in guiding the refolding kinetics of single domains, great emphasis may be placed on preserving their structural integrity. Although C_H_3 alone refolds efficiently, its folding dynamics appears to influence C_H_2 refolding, highlighting interdomain effects. HDX-MS has proven to be a rapid and reliable tool for screening antibodies across a range of conditions, and future experiments could be further designed to screen the stability of specific regions in response to engineered mutations or formulation additives. These findings could inform region-specific engineering strategies aimed at minimizing aggregation driven by partial unfolding within the Fc domain.

## Supplementary Material



## Notes

Additional figures to support the presented data: characterization of the mAb at 1 M Gnd-HCl, DLS after partial unfolding and ON refolding, detailed differential deuterium uptake maps for the whole mAb sequence during the unfolding and refolding experiments, and coverage map of the mAb sequence (PDF)
