# Supplementary material for: Interdomain Interactions Modulate Refolding Kinetics and Aggregation in a Monoclonal Antibody
Source: J Am Soc Mass Spectrom. 2025 Aug 26;36(11):2405–13. doi: 10.1021/jasms.5c00166 (PMC12598857; doi:10.1021/jasms.5c00166)
Supplement: Supplementary file 1 [file js5c00166_si_001.pdf]

Interdomain interactions modulate refolding kinetics and aggregation in a monoclonal antibody

Philipp Trolese[1,2], Andrea Pierangelini[1], Benedetta Fongaro[1], and Patrizia Polverino de Laureto[1,*]

[1]Department of Pharmaceutical and Pharmacological Sciences, University of Padova, Padova 35131, Italy

[2]Department of Neuroscience, Biomedicine and Movement Sciences, University of Verona, Verona 37134, Italy

*corresponding author: Patrizia Polverino de Laureto; patrizia.polverinodelaureto@unipd.it

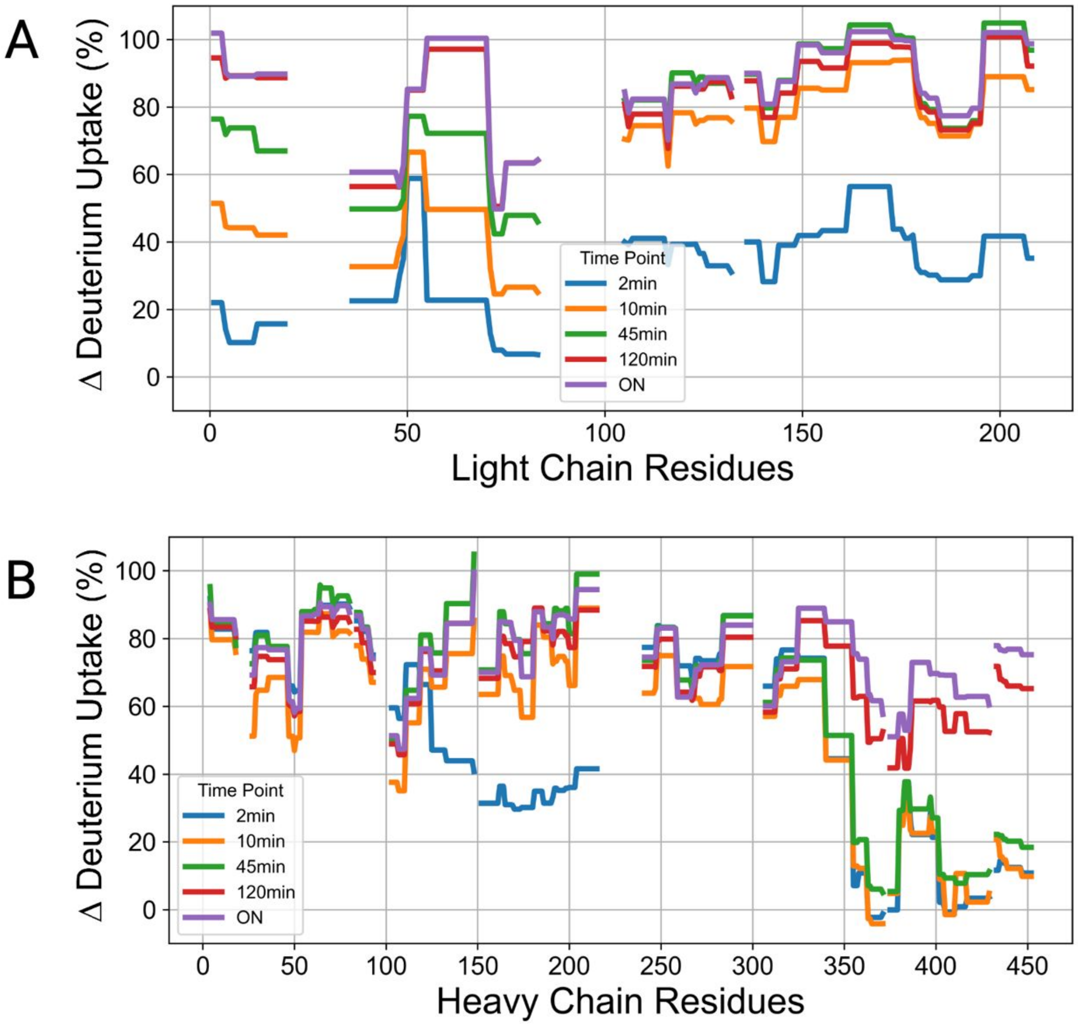

Figure S1. Detailed differential deuterium uptake plot of the whole mAb during unfolding compared to the native state. Each trace represents a specific incubation time: 2 min (blue), 10 min (orange), 45 min (green), 120 min (red), ON (purple).

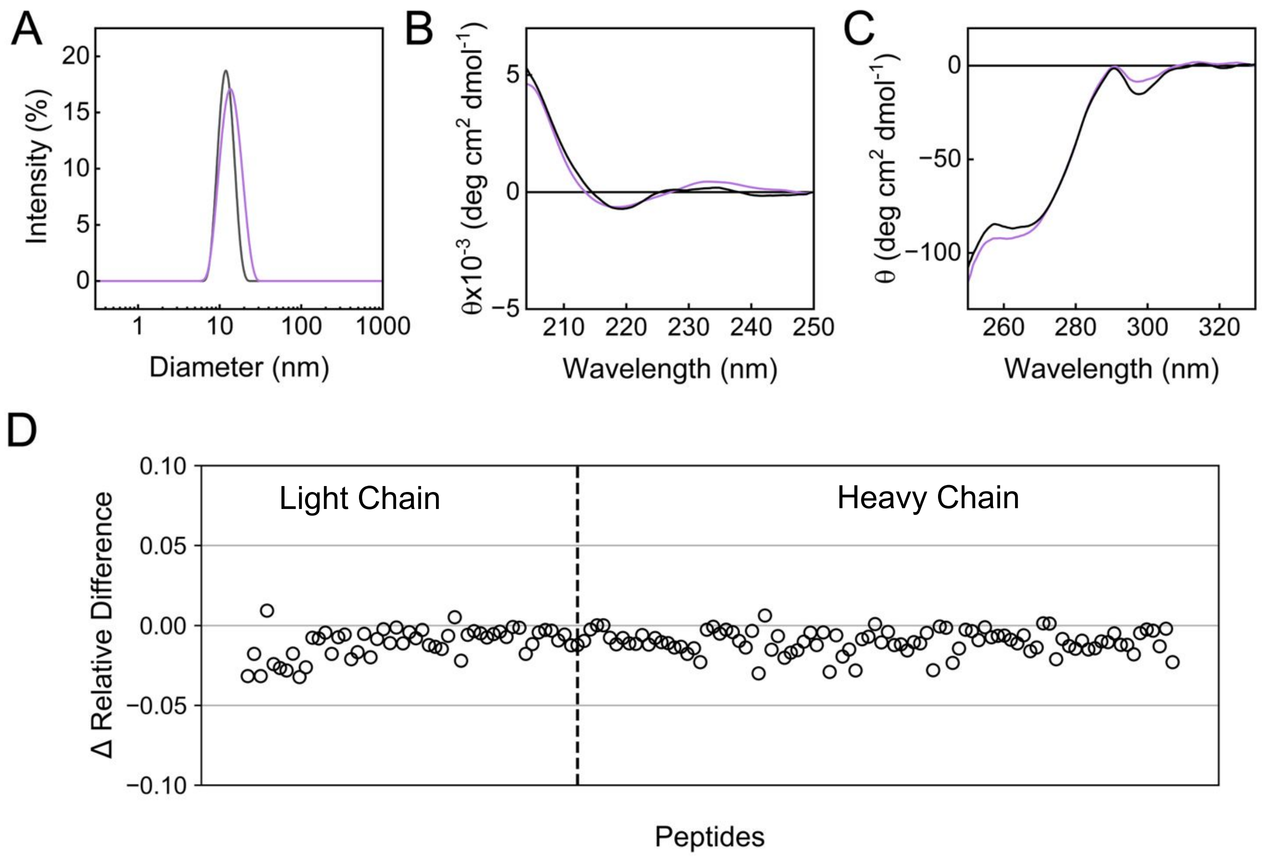

Figure S2. Characterization of Bevacizumab after ON incubation in 1 M Gnd-HCl with DLS (A), far- (B) and near- (C) UV CD and HDX-MS (D). The untreated sample is depicted in black and the incubated sample in purple. DLS shows that no aggregates are present after ON incubation in 1 M Gnd-HCl; CD measurements show that neither secondary nor tertiary structure is lost in the sample. D shows the difference in relative uptake for each of the obtained peptides, i.e. the raw difference in deuterium uptake between the incubated (1 M Gnd-HCl) and the untreated sample (no Gnd-HCl) normalized by the length of each peptide. Only values outside ± 0.05 are significant; therefore, no change in deuterium uptake is observed after treatment.

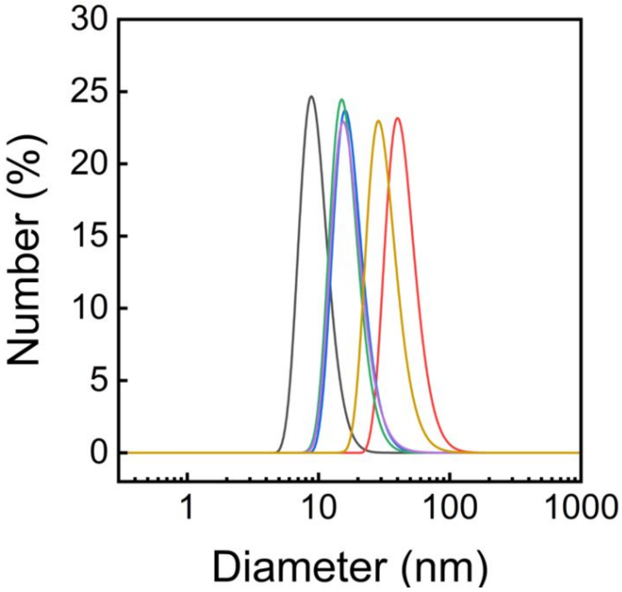

Figure S3. DLS measurements of Bevacizumab incubated in 4 M Gnd-HCl for a specific time (2 min-blue, 10 min-purple, 45 min-green, 120 min-yellow and ON-red, diluted to 1 M Gnd-HCl and incubated ON for the refolding steps.

A

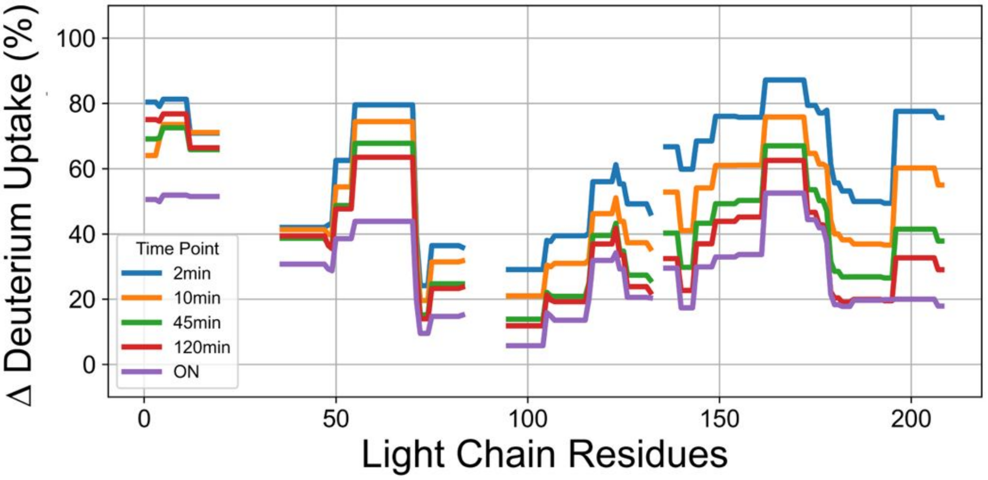

B

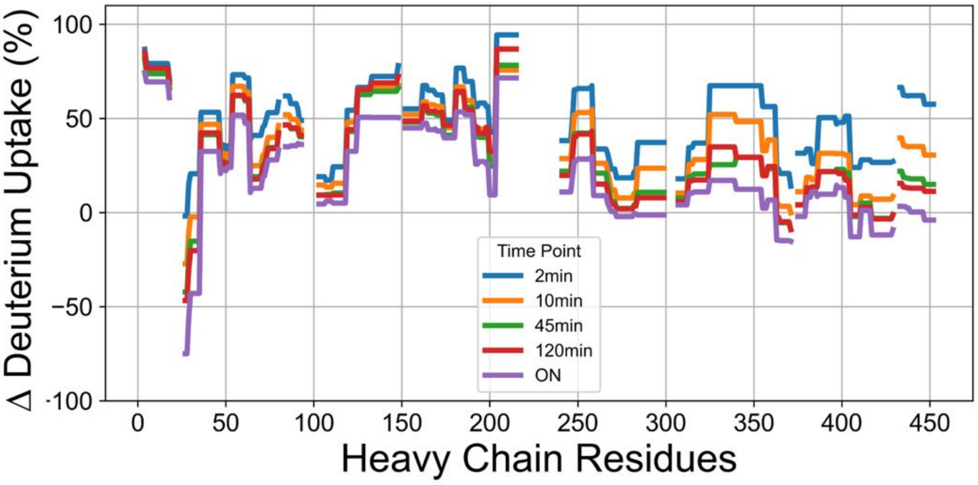

Figure S4. Detailed differential deuterium uptake plot of the whole mAb during refolding from the fully denatured state compared to the native state. Each trace represents a specific incubation time in 1 M Gnd-HCl for refolding: 2 min (blue), 10 min (orange), 45 min (green), 120 min (red), ON (purple).

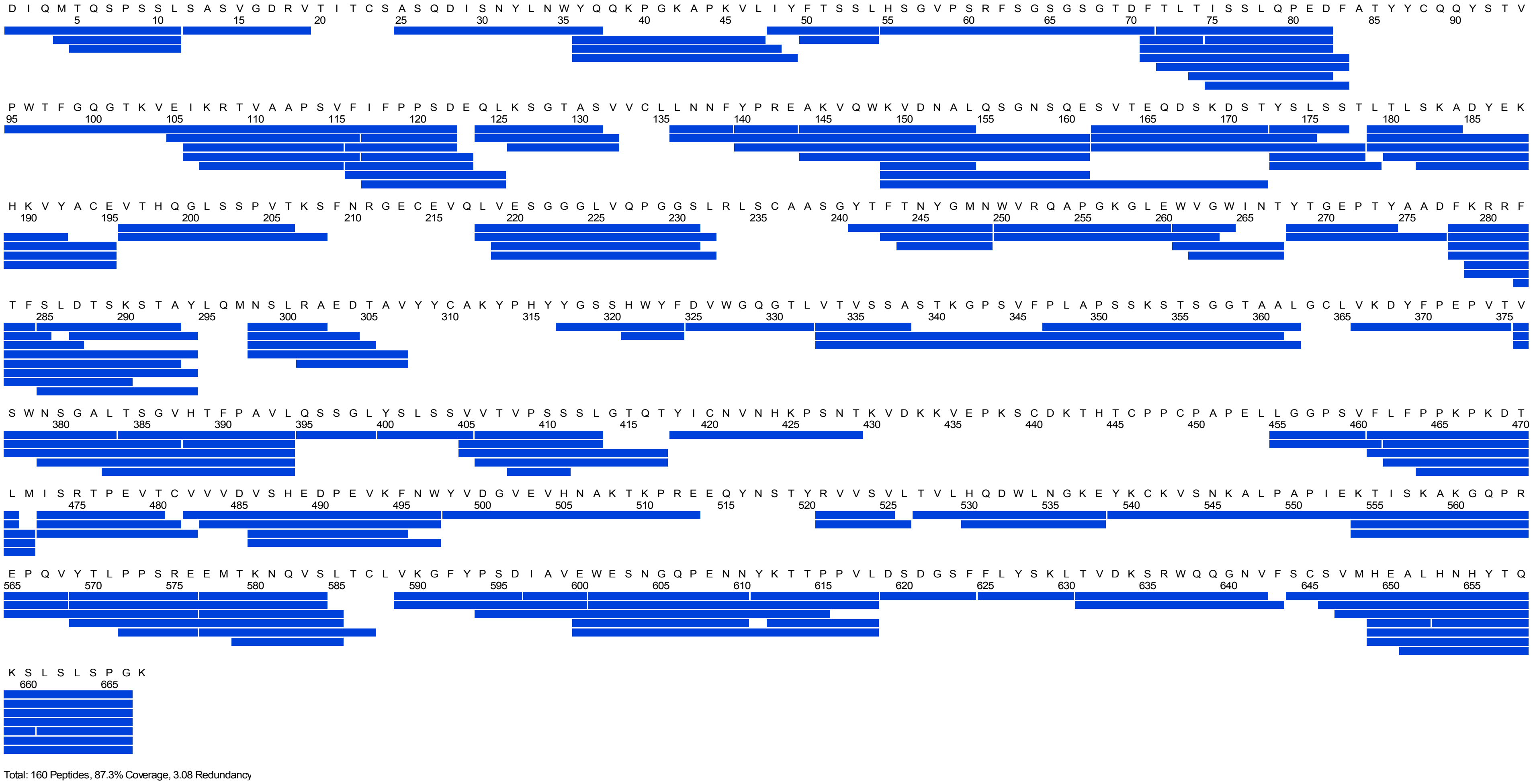

Scheme S1. Protein coverage obtained in the HDX-MS experiments after pepsin cleavage.
